# Environmental Lead Exposure in Polish Children: Blood Lead Levels, Major Sources and Principles of the Lead Poisoning Prevention

**DOI:** 10.1155/S1565363303000268

**Published:** 2003

**Authors:** Dorota Jarosińska, Maja Muszyńska-Graca, Beata Dąbkowska, Joanna Kasznia-Kocot, lwona Sakowska-Maliszewska, Yvonna Woźniakowa

**Affiliations:** Institute of Occupational Medicine and Environmental Health 13 Kościelna Sosnowiec 41-200 Poland

## Abstract

In Poland, children are exposed to lead from the combustion of leaded gasoline and industrial processes. Since the early 1990s, emission levels have declined, and a ban on leaded petrol is anticipated in 2005. Major industrial sources are located in Silesia Province and the copper mining centre (Legnica region). Concerns about, lead exposure in children date back to the 1980s; mean blood lead levels (BILL)reported in children living near lead smelters in Silesia exceeded 20ug/dl. in the 1990s, mean BLLs were decreasing, both in urban children and those living near lead industry. Lower than the CDC action level of 101ug/dl, they were however higher than mean values in children from the other countries, where leaded gasoline had already been banned. Childhood lead poisoning prevention requires a comprehensive approach, involving different sectors. Medical prevention focuses on the early detection of exposed child by the blood lead testing and individual case management. An increasing body of evidence, indicating adverse effects even below the current “safe” level of 101ug/dl, argues for intensification of the primary prevention, which requires legal, economic and technical measures. Public health efforts should contribute to the reduction and elimination of sources of exposure in child’s environment and public education campaigns.


### Environmental Lead Exposure in Polish Children: Blood Lead Levels, Major Sources and Principles of the Lead Poisoning Prevention.

Dorota Jarosińska*, Maja Muszyńska-Graca, Beata Dąbkowska, Joanna Kasznia-Kocot, Iwona Sakowska-Maliszewska, Yvonna Woźniakowa

*Institute of Occupational Medicine and Environmental Health*
*13 Kościelna, 41-200 Sosnowiec, Poland*
*Phone: + 48 32 2660885*
*Fax: + 48 32 2661124*

### ABSTRACT

In Poland, children are exposed to lead from the combustion of leaded gasoline and industrial processes. Since the early 1990s, emission levels have declined, and a ban on leaded petrol is anticipated in 2005. Major industrial sources are located in Silesia Province and the copper mining centre (Legnica region). Concerns about lead exposure in children date back to the 1980s; mean blood lead levels (BLL) reported in children living near lead smelters in Silesia exceeded 20μg/dl. In the 1990s, mean BLLs were decreasing, both in urban children and those living near lead industry. Lower than the CDC action level of 10μg/dl, they were however higher than mean values in children from the other countries, where leaded gasoline had already been banned. Childhood lead poisoning prevention requires a comprehensive approach, involving different sectors. Medical prevention focuses on the early detection of exposed child by the blood lead testing and individual case management. An increasing body of evidence, indicating adverse effects even below the current "safe" level of 10μg/dl, argues for intensification of the primary prevention, which requires legal, economic and technical measures. Public health efforts should contribute to the reduction and elimination of sources of exposure in child's environment and public education campaigns.

* Corresponding author
Institute of Occupational Medicine and Environmental Health
13 Kościelna, 41-200 Sosnowiec, Poland
Phone: + 48 32 2660885
Fax: + 48 32 2661124
Email: d.jarosinska@imp.sosnowiec.pl

### INTRODUCTION

Human exposure to lead remains one of the most serious environmental health problems, especially for children. Particular risk for young children results both from physiological conditions and specific exposure patterns. Children absorb more of the ingested lead than adults do (30-50% and 10%, respectively), have higher metabolic turnover, and their maturing nervous system is highly vulnerable to lead /1/.

Lead can adversely affect human health through the direct inhalation or ingestion of lead contaminated soil, dust or food items. Inhalation is an important route of exposure in occupational setting. For environmental exposures, ingestion plays the major role. In Poland more than 72% of lead uptake is estimated to come from lead deposits in dust and soil; that is consistent with WHO estimates of about 80% /1,2/. Inhalation of airborne lead amounts to as much as 6% of total lead uptake, and uptake with food and water for almost 22% /2/. Children may ingest lead contaminated non-edible particles by normal hand to mouth activity while playing on the dusty playground or in the dirty sandpits, eating or drinking outside without washing hands. Lead containing dust can also be brought home with the working clothes of the adult working with lead.

### ENVIRONMENTAL SOURCES OF LEAD IN POLAND

Main sources of lead emissions in Poland are the combustion of leaded gasoline and industrial processes, in particular non-ferrous metals smelting and combustion processes in the coal-fired power plants.

Over the last decade, lead emissions have been declining, and relative contribution of the sources changed. In the beginning of 1990s, annual lead emission in Poland was almost 1,600 tons and combustion of leaded petrol represented the main source of atmospheric lead emission /2,3/. In the late 1990s, industrial combustion processes in the small and medium sized facilities contributed to almost 40% of air emissions of lead compounds /4/.

Introduction of unleaded gasoline and policies to phase out leaded petrol resulted in a significant decrease of lead emission from the transport sector. In 1992, lead content in the leaded gasoline was reduced from 0.3 to 0.15 g/l, and the limit of 0.013 g/l in unleaded petrol was adopted, following the EU directive /5/. Traffic related lead emission declined from 0.8 tons in 1990 to less than 0.3 tons in 1995, despite substantial increase in the number of motor vehicles in Poland in the last ten years. Figures from 1996 show that it doubled when compared to 1980, and tripled in the case of passenger cars, exceeding 9 million vehicles /4,5/. The share of lead-free gasoline in the total petrol consumption increased gradually, from about 12% in 1990 to 48% in 1996 /4,6/. By the year 2005, a total ban on leaded petrol is expected /7/. Since 1995, installation of catalytic converters is required on new cars /5/.

Lead mining and processing operates almost exclusively in Silesia Province (formerly Katowice Province), the most industrialised and densely populated region of Poland. Although it occupies only 4% of the country's area (11 000 km$^2$), it has more than 4 million inhabitants, 11% of Poland's population /8/. In the early 1990s, about 5 million tons of lead and zinc ores, 4% of the world total, were extracted in the region /4/.

Silesia is also a coal mining and heavy industry centre, with coal-based power, steel, and coke plants. Coal is still a common fuel for individual household heating. Concentration of heavy industry in this most densely populated region of the country resulted in considerable levels of environmental contamination. In the central part of the Province (Upper Silesian Industrial Zone), mean annual concentration of airborne lead in 1990 was 570ng/m$^3$, and lead fallout reached 164mg/m$^2$. In 1997, respective values were 151ng/m$^3$ and 57mg/m$^2$ /9/. The permissible airborne lead concentration (annual mean) is 500 ng/m$^3$, and the lead fallout is 100 mg/m$^2$ per year /10/. The other Polish region with the significant industrial lead emissions is the Lower Silesia Province, former Legnica Province - the centre of copper metallurgy (Fig. 1).

The average lead content in the Polish soils is about 16 ppm, as shown by the national geo-chemical survey. The highest values (up to 17 000 ppm) are found in Silesia Province, with the average 53 ppm; this is partly attributed to the natural occurrence of lead containing ores, and partly to anthropogenic activities /11/. In the Lower Silesia, the highest soil lead levels are found in the vicinity of "Legnica" (up to 970 ppm) and nearby "Glogow" (1963 ppm) non-ferrous metallurgical enterprises /12/. Outside these two provinces, elevated soil lead contents, due to traffic emissions, are reported for the biggest Polish cities (Warszawa, Lodz, Gdansk, Poznan, Szczecin, Bydgoszcz) /12/.

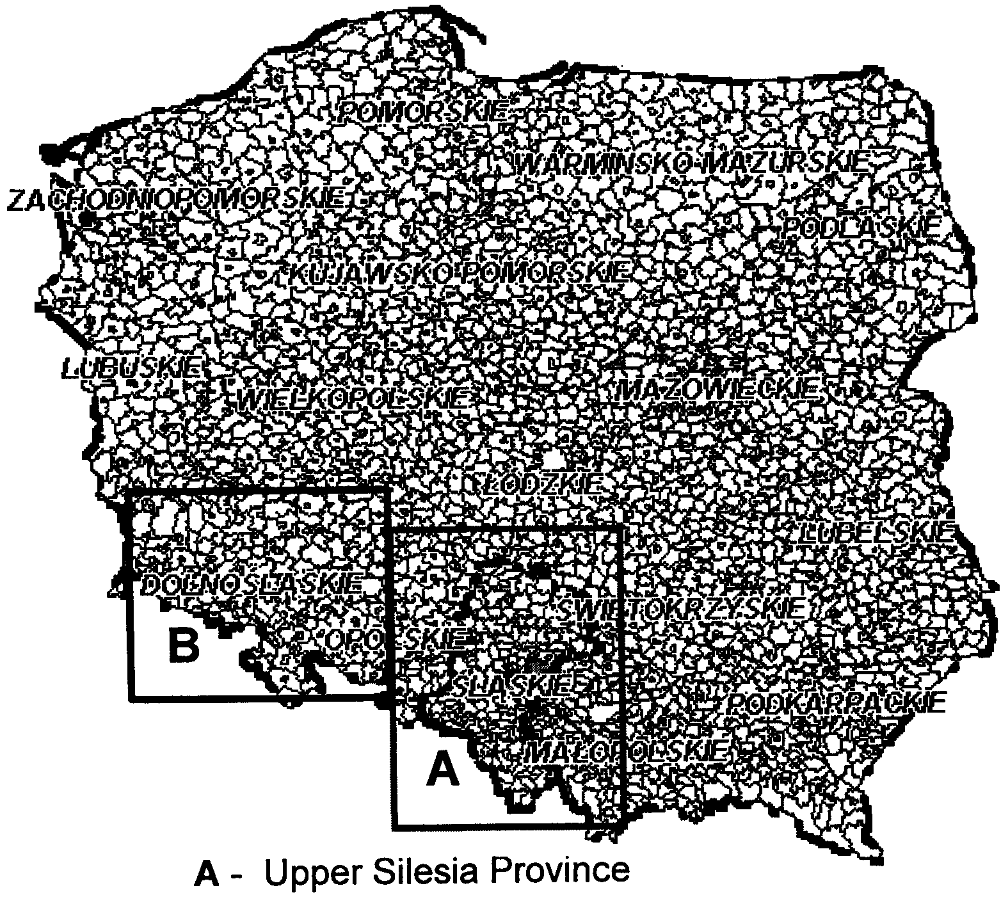

**Fig. 1**: Regions with the main industrial sources of lead in Poland

Lead based paint has never been widely used for house interiors, as Poland signed the ILO convention prohibiting the use of white lead paint already in 1924 /13/. Only anecdotal cases of child's exposure, due to misuse of lead containing paints, have been reported /14/.

Data on lead in food in Poland should be a subject of further investigation, especially with the dynamically changing food market. Lead in drinking water is not generally considered a relevant source of exposure in Poland. In the past, agricultural use of sewage sludge, contaminated with heavy metals, was reported to raise lead content in soil and secondarily contribute to contamination of crops.

### EXPOSURE TO LEAD IN POLISH CHILDREN

Data on blood lead levels in children come from various sources: research projects, monitoring and prevention programmes developed by the research institutes and community based organisations operating in the regions with industrial sources of lead. Data on BLLs are not collected centrally; information is available from the program reports, conference papers, and other publications.

Reports on lead exposure in children date back to the 1980s, when the problem was first recognised in regions with lead industry. The study of school children living within 2 km from the lead smelter in Silesia Province, conducted between 1981-1985 and 1987-1990, revealed mean BLLs of 16,8μg/dl and 25,6μg/dl, almost two times higher than mean values found in the rural children in Silesia /15/. In the beginning of the 1990s, mean BLL near the non-ferrous mill in Silesia was 15μg/dl /16/, and 8 to 10 μg/dl in the area of the copper mill in Legnica Province /after 17/. An epidemiological study conducted between 1992 and 1994 in children aged under 10 years, living in the vicinity of zinc (n=325) and copper (n=282) mills, revealed mean BLLs of 11.4μg/dl and 9.4 μg/dl, respectively; blood lead levels decreased with a distance from the source. Within 5 km from the zinc mill, more than 50% of children had BLL higher than 10μg/dl, and about 25% had BLL higher than 14 μg/dl /17/. In 1995, mean BLL of 13.7μg/dl was reported in the group of children aged 8-15 years (n=158), attending schools located 0.8-7 km from the zinc mill /18/. In the area of direct impact of zinc mill, from 1992 through 1996, mean BLL in six years old children lowered from 11.6μg/dl to 7.2μg/dl, with the proportion of BLL≥10μg/dl decreasing from 69.1 to 18% /19/.

In the late 1980s, mean blood lead levels among urban children aged 6-7 years, living in three central Silesian cities, ranged from 12.3μg/dl to almost 15 μg/dl /15/. A later study (in 1995) in one of these cities, revealed mean BLL 6.2μg/dl in 9 year-old children; 7% had concentrations higher than 10μg/dl /20/. In 1993, mean BLL in the random sample of 431 children living in the adjacent city was 7.9μg/dl, with concentrations exceeding 10μg/dl in 27% (after /20/). The same year, mean BLL in 7 year-old children living in two central Silesian cities (Katowice and Chorzow) were 8.2μg/dl in boys and 7.6μg/dl in girls /21/. Between 1993 and 1999, mean blood lead levels in young Silesian urban children living in the cities within the borders of Katowice Agglomeration ranged from 5.9μg/dl to 8.3μg/dl among the cities, with more than 13% children having elevated BLL /22/. About 14 000 children participated in this population based lead screening and prevention program, conducted by the Institute of Occupational Medicine and Environmental Health.

Results of blood lead testing among more than 30 000 children from Legnica Province - the copper industry centre - from 1995 through 1998 show decrease in mean BLLs from 7,0μg/dl to 5,7μg/dl, with the proportion of children with elevated BLL declining from 16% to 11,6% /23/. Still, higher mean concentrations and higher percentage of BLL exceeding 10μg/dl were found in the areas near the copper mill. Similar trend was shown in a study of over 4 000 children living in one of the towns of Legnica Province (Lubin). Between the years 1992 and 1997, mean BLL in children aged 2-13 years declined from 7,3μg/dl to 3,4μg/dl (with marked decrease staring from 1995), and proportion of BLL>10μg/dl changed from 19,8% to 1,5% /14/.

Information on BLL in children living in other parts of Poland come from relatively few studies. Estimates from 1990 suggested mean BLL in the "clean" areas close to 8,0μg/dl, with proportion of BLL>20μg/dl about 0,5% /24/. In the early 1990s, the reported mean BLL in rural Polish children (aged 7-15 years) was about 7,0 μg/dl /24/.

Between 1992-1994, the epidemiological study of 555 children under 10 years of age living in five big Polish cities with no large lead emitters revealed mean BLL ranging from 2,9 μg/dl to 6,3 μg/dl. The highest value was found in children living in the city centre with heavy traffic; about 10% of children had elevated BLL, and mean lead level was almost two times higher than in the suburban region /17/. Mean BLL reported from Warsaw in 1994, in children aged about 6 years (n= 153) was 4.5μg/dl /after 17/.

The summary presentation of BLL in Polish children living near the industrial sources of lead and urban areas is given in Fig. 2.

Blood lead levels in Polish children have been decreasing over the last decade, both in the urban areas and in locations near to industrial sources of lead. Mean BLL in Polish urban children are lower than the US

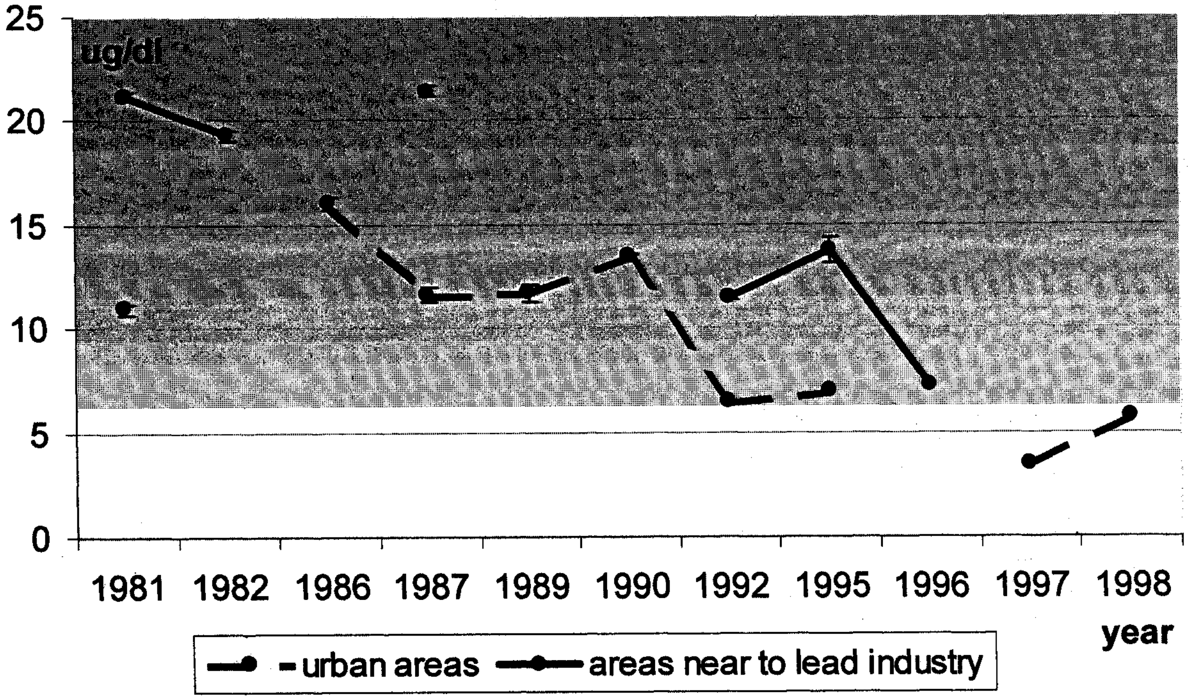

**Fig. 2:** Blood lead levels in Polish children living in urban areas and near industrial sou[illegible]es of lead (14, 15, 17, 18, 19, 20, 21, 23)

Centers for Disease Control and Prevention (CDC) action level of 10μg/dl /25,26/; however, when compared with other countries, they are higher than mean values observed among children from other European countries, where leaded gasoline has already been banned /27,28,29,30,31,32/, and among the US children (aged 3 to 11 years) between 1991-1994 /33/. The levels of exposure among children living near industrial sources of lead in Poland were comparable with those reported from the lead smelter areas in Czech Republic /34/.

A further decrease in the lead exposure of Polish children may be anticipated, following the phase out of leaded gasoline and the declining levels of industrial lead emissions. Monitoring and prevention activities should be continued in the locations with the reported higher mean blood lead levels, in particular in regions with the history of heavy industry and intensive road traffic, such as urban centre of Silesia, and areas close to the lead industry. The assessment of the lead exposure in children living in the big city centres, with rapidly increasing road traffic should also be considered.

### PREVENTION OF CHILDHOOD LEAD POISONING

Childhood lead poisoning prevention requires a comprehensive approach, with the involvement and co-operation of different sectors and actions taken on the central, regional and local levels (Fig. 3). Prevention activities should be undertaken on the individual and population levels, and comprise both primary and secondary measures.

The medical aspect of preventing childhood lead poisoning focuses on the secondary prevention measures, which are: blood lead testing to detect the exposed individuals and the follow up case management, with the range of services depending on the initial blood lead level.

In Poland, the State Sanitary - Epidemiological Commission set recommendations for the secondary prevention of childhood lead poisoning in 1992. Lead screening was recommended for children aged 7-10 children living near the industrial sources, with CDC guidelines in children with elevated BLL /2/. There are no specific recommendations for urban children. A 50% reduction in the proportion of urban children with elevated blood lead levels is defined as one of the strategic goals in the Polish National Health Program for the years 1996-2005 /35/.

The range of follow-up services offered to children with elevated blood lead levels in Poland may differ among the regions, depending on the local expertise.

The Institute of Occupational Medicine and Environmental Health in Sosnowiec has extensive experience in childhood lead poisoning prevention activities. The large-scale, population based program, with more than 14 000 participating children, initiated by the Institute of Occupational Medicine and Environmental Health in 1993, comprised three main elements:

- blood lead testing in young urban children
- identification of children with elevated blood lead level
- provision of individual case management to the lead exposed child

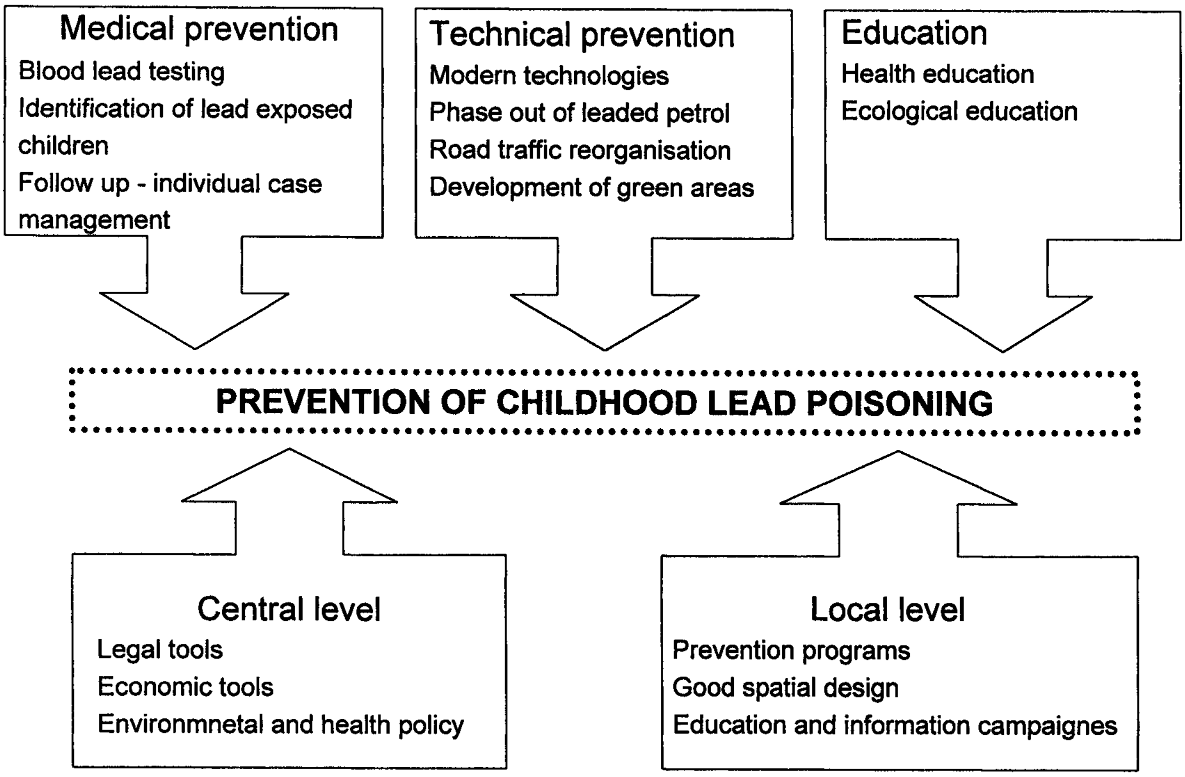

**Fig. 3**: Key elements of childhood lead poisoning prevention

Children with blood lead levels exceeding 10μg/dl were referred to the Department of Environmental Medicine at IOMEH for individual assessment and case management. An algorithm of the medical case management, developed in our Department, included blood count, assessment of mineral status (calcium, magnesium, iron), psychological testing and specialist consultations as decided by the paediatrician. This algorithm will be soon be evaluated with reference to the recently published CDC recommendations on the management of elevated blood lead levels among young children /36/.

Experiences form the large-scale screening program and individual consultations of the lead exposed children were further used to elaborate a set of educational materials on lead poisoning prevention. The contents of the individually designed leaflets and brochures were differentiated, to make them most useful and attractive for the addressees, who are: the children and their parents, teachers, physicians (primary care providers) and nurses, workers occupationally exposed to lead, as well as the representatives of local administration. As an example, the leaflet for children is presented in Fig. 4.

At present, blood lead concentration of 10μg/dl is used as a level of concern,triggering public health programs aiming at early detection and treatment of lead exposed children /25,26/. However, there is an increasing body of evidence indicating adverse neurodevelopmental effects even below this "safe" level, and chelation therapy was shown to be ineffective to prevent the neurotoxic harm due to moderate lead exposure in young children /37,38/.

The current knowledge on the lead toxicity and observed changes in the level of lead exposure in children argues for a change in approach towards the management of childhood lead toxicity, with emphasis on the importance of primary prevention. Protecting children from the exposure to lead requires legal, economic and technical measures. Public health efforts should contribute to the reduction and elimination of sources of exposure in child's environment and public education campaigns.

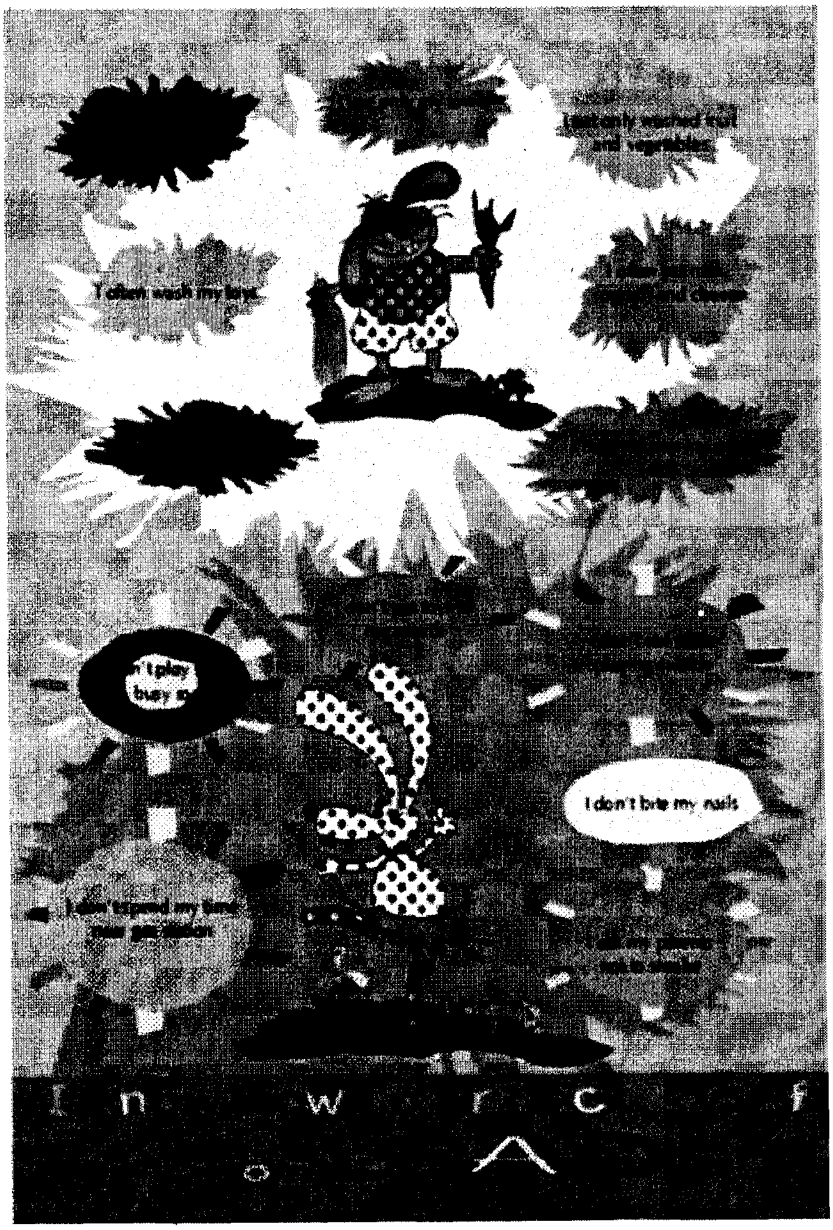

**Fig. 4:** The educational leaflet for children (design: E. Galkowska)

### ABBREVIATIONS USED

| | |
|---|---|
| BLL | BLOOD LEAD LEVEL |
| CDC | CENTERS FOR DISEASE CONTROL AND PREVENTION |
| WHO | WORLD HEALTH ORGANISATION |

### ACKNOWLEDGEMENTS

D. Jarosinska would like to express thanks to Dr Walter Rogan from the Epidemiology Branch, National Institute of Environmental Health Sciences (NIEHS), RTP, US for the stimulating discussions during her Fulbright scholarship at NIEHS.

The help of Dr Shyamal Peddada, from the Biostatistics Branch, National Institute of Environmental Health Sciences RTP, US in developing a graph for Fig. 2, is gratefully acknowledged.

### REFERENCES

1. Environmental Health Criteria 165. WHO/IPCS, Geneva (1995)
2. Toxicological Commission of the Sanitary and Epidemiological Council. *Medycyna Pracy*, **6, Suppl 1**, 5 (1993) (in Polish)
3. T. Dutkiewicz, J. Swiatczak, *Medycyna Pracy*, **6**, Suppl 1, 53 (1993) (in Polish)
4. State Inspection of Environmental Protection, Report on the state of the environment in Poland (1998). Available: http://www.mos.gov.pl/soe/index.html (accessed 22 October 2002)
5. Sofia Initiative on Local Air Quality. Phase-out of leaded gasoline. Regional Environmental Centre for Central and Eastern Europe, Szentendre (1998)
6. Pan-European Strategy to Phase-out Leaded Petrol. 4th Ministerial Conference: Environment for Europe. Arhus, Denmark (1998)
7. Polish Council of Ministers, II National Environmental Policy of Poland (2000) Available:http://www.mos.gov.pl/mos/publikac/Raporty_opracowania/pol_ekoll_nowy/index.htm (accessed 10 September 2002)
8. UNDP Project on Urban Environmental Management and Sustainable Development in the Katowice Agglomeration. Environmental Profile of Katowice Agglomeration (1999)
9. Provincial Sanitary Epidemiological Station in Katowice (WSSE). Ambient air pollution in Katowice Province in 1998 (1999) (in Polish)
10. Minister of Environmental Protection, Natural Resources and Forestry. Regulation of the 28 April 1998 on the permissible levels of concentration of pollutants in ambient air (in Polish). Available: http://www.mos.gov.pl (accessed 20 September 2002)
11. Geo-chemical atlas of the Upper Silesia. The National Geological Institute, Warsaw, Poland (1995)
12. Geo-chemical atlas of Poland. The National Geological Institute, Warsaw, Poland (1995)
13. E.K. Silbergeld, *Annu Rev Public Health*, **18**, 187 (1997)
14. R. Andrzejak, L. Gruszczynski, K. Lisowska, *Proceedings of the Conference of the Foundation for the Children in the Copper Basin* (1997) (in Polish). Available: http://www.fundacja.pl (accessed 22 June 2002)
15. J. Grabecki, *Medycyna Pracy*, **6**, Suppl 1, 85 (1993) (in Polish)
16. K. Osman, L. Bjorkman, B. Lind, M. Nordberg, *Int J Environ Health Res*, **2**, 212 (1992)
17. M. Jakubowski, M. Trzcinka-Ochocka, G. Razniewska, JM. Christensen, A. Starek, *Int Arch Occup Environ Health*, **68**, 193 (1996)

18. J. Chlopicka, Z. Zachwieja, P. Zagrodzki, J. Frydrych, P. Slota, M. Krosniak, *Biological Trace Element Research*, **62**,229 (1998)
19. M. Dumienski, Report of the Foundation for Children "Miasteczko Sl.", Miasteczko Sl (1996) (in Polish)
20. K. Osman, *Int Arch Occup Environ Health*, **71**,180 (1998)
21. J.E. Zejda, J.A. Sokal, J. Grabecki, Z. Panasiuk, M. Jarkowski, M. Skiba, *Cent Eur J Public Health*, **3**, 92 (1995)
22. D. Jarosinska, J. Kasznia-Kocot, M. Muszynska, *Metal Ions Biology and Medicine*, John Libbey Eurotext, Paris **5**, 332 (1998)
23. H. Strugala-Stawik, D. Dembicka, B. Pastuszek, *Proceedings of the Conference of the Foundation for the Children in the Copper Basin* (1998) (in Polish). Available: http://www.fundacja.pl (accessed 22 June 2002)
24. T. Dutkiewicz, E. Kulka, *Medycyna Pracy*, **6**, Suppl 1, 77 (1993) (in Polish)
25. Centers for Disease Control and Prevention (US CDC): Preventing Lead Poisoning in Young Children (1991)
26. Centers for Disease Control and Prevention (US CDC): Screening Young Children for Lead Poisoning: Guidance for State and Local Public Health Officials (1997)
27. J. Begerow, I. Freier, M. Turfels, U. Krämer, L. Dunemann, *Int Arch Occup Environ Health*, **66**, 243 (1994)
28. A. Furman, M. Laleli, *Sci Total Environ* , **234**, 37 (1999)
29. J. O'Donohoe, S. Chalkley, J. Richmond, D. Barltrop, *Clin Sci*, **95**, 219 (1998)
30. A. Pönka, *Sci Tot Environ*, **219**, 1 (1998)
31. M. Schumacher, M. Bellés, A. Rico, JL. Domingo, J. Corbella, *Sci Total Environ*, **184**, 203 (1996)
32. U. Strömberg, A. Schütz, S. Skerfving, *Occup Environ Med*, **52**, 764 (1995)
33. J.L. Pirkle, R.B. Kaufmann, D.J. Brody, T. Hickman, E.W. Gunter, D.C. Paschal, *Environ Health Perspect*, **106**, 745 (1998)
34. M. Cirkt, Z. Smerhovsky, K. Blaha, J. Nerudova, V. Sediva, J. Fornuskova et al., *Environ Health Perspect* ,**105**, 406 (1997)
35. Intersectoral Task Force for the National Health Program, 1996-2005. Ministry of Health and Social Welfare, Warsaw, Poland (1996). Available: www.medstat.waw.pl (accessed 02 October 2002)
36. Centers for Disease Control and Prevention (US CDC): Managing Elevated Blood Lead Levels among Young Children: Recommendations from the Advisory Committee on Childhood Lead Poisoning Prevention (2002)
37. B.P. Lanphear, K. Dietrich, P. Auinger, C. Cox, *Public Health Rep*, **115**, 521 (2000)
38. W.J. Rogan, K.N. Dietrich, J.H. Ware, D.W. Dockery, M. Salganik, J. Radcliffe et al., *N Engl J Med*, **344**, 1421 (2001)

